# Living in stressful neighbourhoods during pregnancy: an observational study of crime rates and birth outcomes

**DOI:** 10.1093/eurpub/ckw131

**Published:** 2016-08-29

**Authors:** Tom Clemens, Chris Dibben

**Affiliations:** School of Geosciences, University of Edinburgh, Edinburgh, UK

## Abstract

**Background:** Patterns of adverse birth outcomes vary spatially and there is evidence that this may relate to features of the physical environment such as air pollution. However, other social characteristics of the environment such as levels of crime are relatively understudied. This study examines the association between crime rates and birth weight and prematurity. **Methods**: Maternity inpatient data recorded at birth, including residential postcode, was linked to a representative 5% sample of Scottish Census data and small area crime rates from Scottish Police forces. Coefficients associated with crime were reported from crude and confounder adjusted models predicting low birth weight (< 2500 g), mean birthweight, small for gestational age and prematurity for all singleton live births. **Results**: Total crime rates were associated with strong and significant reductions in mean birth weight and increases in the risks of both a small for gestational age baby and premature birth. These effects, with the exception of prematurity, were robust to adjustment for individual characteristics including smoking, ethnicity and other socio-economic variables as well as area based confounders including air pollution. Mean birth weight was robust to additional adjustment for neighbourhood income deprivation. **Conclusion**: The level of crime in a mother’s area of residence, which may be a proxy for the degree of threat felt and therefore stress experienced, appears to be an important determinant of the risk of adverse birth outcomes.

## Introduction

Birth outcomes including birthweight and preterm birth have been shown to be associated with a variety of health outcomes,^1^^,^^2^ cognitive development,^3^ educational attainment^4^ and psychiatric disorders.^5^ These birth outcomes have also been shown to be associated with a range of social, environmental and health factors including social class, smoking and drinking and ethnicity.^6^

There is growing evidence that the neighbourhoods in which individuals live may exert independent effects for health more generally^7^ and adverse birth outcomes more specifically.^8^ Indices of multiple deprivation using domains including education, housing, employment and health^9^ have been used to capture area based material disadvantage^8^^,^^10^ or theorise and test particular candidates including variations in social support^11^ and access to care^12^ in different neighbourhoods. Furthermore, a growing literature has developed in environmental epidemiology which has shown that ambient background levels of pollutants such as nitrogen dioxide, particulate matter less than 10 microns and sulphur dioxide have all been linked to increased risks of low and very low birth weight and preterm babies as well as lower mean birth weight.^13^^,^^14^

Another possible explanation of the spatial variation in birth outcomes is that certain local neighbourhoods are more stressful environments in which to live during pregnancy. There are plausible hormonal pathways linking the two including levels of certain hormones such as placental corticotropin-releasing hormone (CRH) and adreno-corticotropin hormone (ACTH). Both are important mediators in the pathway towards elevated risks of experiencing both premature birth and small for gestational age (SGA) baby^15^^,^^16^ as well as being an important component in the physiological adaptation and response to chronic stress exposure.^16^^,^^17^ Furthermore, it has long been established that stressors originating in the local environment are an important component of an individual’s total stress load.^18^

A fairly large number of studies investigating stress and adverse birth outcomes have focused on exposure to acute stressors finding associations with events such as earthquakes,^19^ hurricanes,^20^ conflict and civil unrest^21^ and incidents of terrorism.^22^ However, while these studies are useful in determining which periods of pregnancy are particularly susceptible to stress, they tell us little about possible effects associated with chronic exposure.

Other studies focused on chronic contextual stressors such as incidents, rates and perceptions of crime in local neighbourhoods. The possibility of crime rates explaining spatial patterns in general health outcomes has been explored^23–25^ and more recently has been extended to associations between adverse birth outcomes and perceptions of crime,^26^ proximal measures relating residential distance from crime events^27^ and area based crime rates.^27–29^ Interestingly, the evidence from these studies seems to suggest that crime is most strongly associated when it is measured at an aggregated area level rather than as the distance of crime events from the mother. This suggests that it may not be the effect of being a victim of crime, which after all will be a rare occurrence, that is important but more that crime rates capture latent characteristics of those neighbourhoods in which exposed pregnant mothers live. This might relate to a feeling of potential threat and therefore higher levels of stress.

However, there remains a lack of consistent international evidence linking local environment sources of chronic or ambient stressors such as crime rates to adverse birth outcomes that is adequately adjusted for potentially confounding effects such as air pollution for example. In this study we test for an association between potentially stressful neighbourhood environments in Scotland, measured through small area based total crime rates in mother’s place of residence, and various adverse outcomes including prematurity and foetal development. We hypothesise that the levels of recorded crime in the mother’s immediate local area of residence will be associated with outcomes at birth.

## Methods

### Study population and birth outcomes data

The Scottish Longitudinal Study (SLS), a 5% sample of the Scottish population, linking census records and a number of other administrative data sets,^30^ was used for this study. Point of delivery information (estimated gestational age, birth-weight, whether the baby was born in a singleton or multiple birth and its gender) and maternal characteristics during the pregnancy (mothers age and usual place of residence, smoking behaviour during pregnancy, parity and occupation of both the mother and father (where present) was obtained from record linkage to maternity hospital inpatient data from the Scottish Morbidity Record (SMR) 02.

The sample was restricted to singleton births (born between 1994 and 2008 inclusive), those that contained complete, non-missing information and those with a valid postcode for the mother’s usual place of residence when the birth was registered. We derived the following outcomes; risk of moderately (32–36 weeks) and very (< 32 weeks) preterm birth, risk of small for gestational age (SGA—risk of birth in the lightest decile of sex and gestational age specific birthweight distributions), risk of low birthweight (LBW < 2500 g) and mean birth-weight. For the models predicting prematurity and SGA all singleton live births were included but for the other outcomes premature births (less than 37 weeks) were excluded.

### Exposure assessment

The crime domain of the Scottish Index of Multiple Deprivation (SIMD) was used to estimate mothers’ ‘exposure’ and was linked to the main sample through residential postcode. The SIMD crime domain records incidents of crime from police forces across Scotland. This measure expresses the total number of crimes of violence; domestic house breaking; drugs offences; minor assault; and vandalisms as a rate per 10 000 population at a datazone level (6505 in Scotland with each zone designed to contain between 500 and 1000 households). Given the relatively higher frequency and spatial correlation of vandalism, drug offences and minor assaults, this measure will tend to be high in areas where these events are high. The measure is therefore capturing areas that are visibly confrontational and potentially violent. Previous studies have demonstrated that these types of areas in particular have a direct and independent influence on individuals fear of crime.^31^^,^^32^

We used the 2006 and 2009 releases of the SIMD which contain crime statistics from 2004 and 2007/2008, respectively. Exposure to levels of crime was determined by taking the centroid of the maternal residential postcode recorded in the maternity hospital record and linking this to the corresponding datazone. Datazones in city centres were excluded as they contained disproportionately higher rates of crime associated with night-time type urban activity.

### Covariates

Confounding is a problem when analysing associations between a spatial exposure such as area crime rates and birth outcomes because the latter is strongly associated with other social and residential characteristics of the mother that may also be risk factors. The relative importance of individual and area factors and their association with health has been a matter of debate for many decades, both generally^33^ and in the context of the outcomes of pregnancy.^8^ In this study, we adjusted for a wide range of socio-economic factors from the census and birth record including, social class, lone parenthood, mother’s education and an estimated measure of income based on occupation.^34^ Additional adjustment was also made for season and year of birth (to control for temporal trends in birth outcomes) and modelled small area estimates of air pollution [sulphur dioxide (SO_2_), nitrogen dioxide (NO_2_) and particulate matter smaller than 10 microns (PM_10_)].

Finally, adverse birth outcomes such as infant mortality are associated with area deprivation^10^ and so are crime rates.^25^ Thus, we also considered the sensitivity of the results to the inclusion of an area based measure of low income. This was obtained from the income domain of the SIMD which ranks areas (datazones) on the basis of the proportion of people in that area who are receiving, or are dependent on, benefits related to low or no income. This variable was included to test whether there might be other unmeasured socio-economic confounders of the crime–birth outcome relationship.

### Statistical methods

Multilevel logistic, linear and multinomial regression models were used to explore the relationship between different birth outcomes and neighbourhood crime rates. The crime variable was log-transformed because the various crime measure distributions were highly positively skewed. We tested a number of different functional forms of varying degrees, including non-integer polynomial functions^35^ and found the natural log to be the best fit. We used multilevel models to correct for spatial dependency. Individual mothers were assigned to level 1 and her datazone of residence to level 2. All models were fitted in STATA version 11 using the xtlogit, xtreg and mlogit with survey estimation.

## Results

Table 1 presents descriptive statistics for the analysis sample and shows the distribution of both outcome and confounding variables that are used in the analysis. Table 2 describes the distribution of the crime rates in both the term births and all births samples. Table 3 shows unadjusted crude model coefficients with crime as the only predictor and indicates that crime is a significant predictor of mean birth weight, risk of low birth weight and small for gestational age and risk of severe but not moderate prematurity. In the models that adjust for confounding factors, these effects were attenuated with only the coefficient for mean birth weight and the odds ratios for the risk of small for gestational age and low birth weight remaining significant. These effects correspond to absolute reductions in mean birth weight of approximately 90 g and increases in the risk of small for gestational age of approximately 62% and low birth weight of 119% between the areas of lowest and highest rates of crime in Scotland.

**Table 1:** Descriptive statistics for the SLS sample for both outcomes and covariates

	Term births		Preterm births	
Categorical variables	*N*	%	*N*	%
Low birthweight (< 2500g)				
Yes	455	2.08	n/a	
No	21,426	97.92	n/a	
Prematurity				
Less than 32 weeks	n/a		193	0.83
Between 32 and 36 weeks inclusive	n/a		1,049	4.54
Greater than 36 weeks	n/a		21,881	94.63
Small for gestational age				
No	n/a		20,921	90.48
Yes	n/a		2,202	9.52
Social class				
Professional	935	4.27	972	4.20
Managerial and technical	5,814	26.57	6,090	26.34
Skilled non-manual	7,533	34.43	7,970	34.47
Skilled manual	1,857	8.49	1,961	8.48
Partly skilled	3,499	15.99	3,740	16.17
Unskilled	934	4.27	993	4.29
Armed forces	<15	<0.1	<15	<0.1
Unemployed	1,302	5.95	1,390	6.01
Parity				
Multiparous	12,570	57.45	13,176	56.98
Nulliparous	9,311	42.55	9,947	43.02
Smoker during pregnancy				
No	16,710	76.37	17,546	75.88
Yes	5,171	23.63	5,577	24.12
Ethnicity				
Non-South Asian	21,612	98.77	22,841	98.78
South Asian	269	1.23	282	1.22
Mothers age at delivery				
17–18	546	2.50	579	2.50
19–23	3,070	14.03	3,257	14.09
24–28	5,986	27.36	6,321	27.34
29–33	7,301	33.37	7,709	33.34
34–38	4,077	18.63	4,290	18.55
39+	901	4.12	967	4.18
Education				
None	3,008	13.75	3,226	13.95
O' grade, Standard grade or equivalent	4,921	22.49	5,236	22.64
Higher, 'A' level, AS level or equivalent	1,670	7.63	1,767	7.64
GSVQ/SVQ Level 1 or 2 or equivalent	4,179	19.10	4,420	19.12
GSVQ/SVQ Level 3, ONC, OND or equivalent	1,068	4.88	1,128	4.88
HNC, HND, SVQ Level 4 or 5 or equivalent	2,057	9.40	2,148	9.29
First degree or higher degree or equivalent	1,714	7.83	1,786	7.72
Professional qualifications or equivalent	3,264	14.92	3,412	14.76
Season of birth				
Winter	5,094	23.28	5,412	23.41
Spring	5,538	25.31	5,840	25.26
Summer	5,623	25.70	5,966	25.80
Autumn	5,626	25.71	5,905	25.54
Lone mother				
No	20,305	92.80	21,419	92.63
Yes	1,576	7.20	1,704	7.37
SIMD income deprivation quintiles				
Quintile 1	4,980	22.76	5,284	22.85
Quintile 2	4,492	20.53	4,772	20.64
Quintile 3	4,248	19.41	4,488	19.41
Quintile 4	4,026	18.40	4,243	18.35
Quintile 5	4,135	18.90	4,336	18.75

**Continuous variables**	**Mean**			

Birth weight (g)	3481.30		n/a	
Estimated weekly wage (£)	353.34		352.27	
Gestational age (weeks)	39.68		39.37	
Air pollution				
Particulate matter < 10 microns (µg/m^3^)	13.30		13.30	
Sulphur dioxide (µg/m^3^)	5.41		5.42	
Nitrogen dioxide (µg/m^3^)	17.47		17.48

**Table 2:** Summary measures of log crime rates (per 10 000 population) for recorded births (1994–2008) in datazones in Scotland

	*N*	Mean	SD	Min	Max	Range
Term births only						
Total crime rate	21881	6.00	0.86	3.64	9.20	5.56
Preterm births						
Total crime rate	23123	6.00	0.86	3.64	9.20	5.56

**Table 3:** Associations between log transformed rates of total crime in maternal datazone of residence and birth outcomes

	Unadjusted		Adjusted^a^	
	Effect size^b^	CI (p < 95%)	Effect size^b^	CI (*P* < 95%)
Continuous birthweight (linear coefficient)	−58.07***	−66.27, −49.86	−16.00***	−24.66, −7.34
Risk of LBW < 2.5kg (odds ratio)	1.46***	1.31,1.64	1.15**	1.01, 1.31
Risk of very or moderately preterm birth				
Moderately preterm 32–37 wks (relative risk ratio)	1.11***	1.04,1.20	1.05	0.96, 1.13
Very preterm <32 weeks (relative risk ratio)	1.04ns	0.88, 1.23	0.91ns	0.75, 1.09
Risk of small for gestational age (odds ratio)	1.32***	1.25, 1.39	1.09***	1.02, 1.16

ns, not significant.

a Adjusted models control for pollution, social class, parity, estimated income, ethnicity, lone parenthood, smoking, maternal age, maternal education, season and year of birth.

b Effect sizes (coefficient, odds ratio or relative risk ratio where appropriate) report change in outcome associated with a one unit increase in log crime rates.

^***^P < 0.01, ^**^P < 0.05, *P < 0.1.

When testing the sensitivity of these results to adjustment for area income deprivation (Table 4), many of the effects attenuated. The biggest attenuation was for the odds ratios of LBW and SGA which were no longer statistically significant. Though not as marked, there was also attenuation of the mean birth weight effect but this effect remained significant at 11 g per 1 unit increase in log scaled crime rates. This estimate translates into a reduction in mean birth weight of 62 g between those areas in Scotland with the lowest and highest rates of crime in Scotland adjusting for confounding factors.

**Table 4:** Sensitivity of the associations between total crime counts in maternal datazone of residence and birth outcomes to adjustment for area income deprivation^a^

	Effect size^b^	CI (*P* < 95%)
Continuous birthweight (linear coefficient)	−11.01**	−21.58, −0.44
Risk of LBW < 2.5 kg (odds ratio)	1.01 ns	0.86, 1.19
Risk of very or moderately preterm birth		
Moderately preterm 32–37 weeks (relative risk ratio)	1.06 ns	0.96, 1.17
Very preterm <32 weeks (relative risk ratio)	0.91 ns	0.73, 1.15
Risk of small for gestational age (odds ratio)	1.02 ns	0.95, 1.10

ns, not significant.

a Adjusted for pollution, social class, parity, estimated income, ethnicity, lone parenthood, smoking, maternal age, maternal education, season of birth, year of birth and quintiles of area income deprivation.

b Effect sizes (coefficient, odds ratio or relative risk ratio where appropriate) report change in outcome associated with a one unit increase in log crime rates.

^***^P < 0.01, ^**^P < 0.05, *P < 0.1.

## Discussion

From a nationally representative sample of pregnant mothers in Scotland, this study has found that area rates of crime in the maternal area of residence are associated with large and significant reductions in mean birth weight and increases in the risks of both a small for gestational age baby and premature birth. These associations, with the exception of prematurity, were robust to adjustment for a range of individual characteristics including smoking and ethnicity as well as area based estimates of pollution concentrations. However, with adjustment for area income deprivation only mean birth weight was significantly associated with area crime rates.

Our findings appear to broadly corroborate results from the relatively few previous studies in this area.^29^^,^^36^^,^^37^ Messer *et al.*^28^ tested both spatial proximity to and overall area rates of violent crime and found no effect when using the former but reported a risk of low birthweight odds ratio of 1.5 for areas in the highest crime tertile compared to the lowest for non-Hispanic white women. These effects appear smaller in magnitude than those reported in this study. They also reported the strongest associations with foetal growth rather than prematurity; which is supported by our findings and other reported evidence.^27^

Only one other study examined different crime types and was restricted to an examination of the risk of preterm birth stratified by race.^28^ They found that none of the crime measures (violent, theft, property and vice crimes) showed any significant association with prematurity after adjustment for other characteristics. This supports the general finding of this paper that prematurity appears more weakly associated with area crime rates than measures of foetal development.

There is evidence to suggest that chronic rather than acute experiences are the more epidemiologically important forms of stress which is perhaps due to the accumulation of physiologically damaging effects over longer periods of time. This process adds to an individual’s ‘allostatic load,’^38^ the degree to which individuals are affected by the wear and tear that occurs throughout their lifetime, particularly as the result of the body’s hormonal and neurotransmitter mediators that ready the body for immediate threats. If these systems are activated on a chronic basis (e.g. through the exposure to a feeling of threat when a person leaves their house) the body is less able to return to a normal state with long term impacts on an individual’s health. The continued exposure to environments containing a greater level of perceived threat such as rates of crime represents a plausible stressor on the pathway to increases in allostatic load and therefore adverse birth outcomes.

In the main models in this study, we did not, unlike other studies,^27^^,^^28^ adjust for other area characteristics (e.g. area rates of individuals on low incomes), apart from a measure of air pollution. However, the results from the sensitivity analysis which show an attenuating effect of introducing area income income deprivation is worthy of further discussion. Given the high degree of collinearity between this measure and area crime rates, it is unsurprising that the majority of the effects attenuated when including adjustment for the former. Importantly, the magnitude of this attenuation is greater for the LBW and SGA models than for mean birth weight. This would appear to suggest that the independent negative effects associated with crime are restricted to the main portion of the covariate adjusted birth weight distribution rather than the extreme left portion of both the main distribution and the gestational age and gender specific distributions. In other words, it appears that area crime rates are more highly correlated with area income deprivation amongst LBW and SGA babies than those whose weight lies closer to the mean of the overall distribution.

There are a number of possible explanations or interpretations for this effect. It could be that there is an upper threshold limit for the effect of external environmental exposures beyond which additional restriction to a baby’s attainable weight is impossible. This interpretation is premised on the notion that baby weights in the lower ranges of the birth weight distribution are likely to be to mothers who been exposed to multiple individual and environmental risk factors which act in combination to greatly restrict the baby’s attainable weight. Amongst these babies, it is possible that exposure to additional environmental exposures such as neighbourhood crime rates, has a reduced effect because their attainable weight is sufficiently low that greater restriction is impossible. Thus, in the sensitivity models, the crime variable competes with the area income deprivation variable for this limited weight restricting environmental effect. Conversely, babies whose attainable weight is closer to the overall mean of the birth weight distribution may be more susceptible to additional environmental insults, particularly the chronic forms of stress that may be associated with living in environments characterised by high rates of crime. The higher attainable weights in this area of the distribution allows for greater scope for additional exposures to affect birth weight with this greater variation allowing crime to retain predictive power when including the collinear predictor of area income deprivation. This might explain why the effect of exposure to areas with high crime rates is greater for mean birth weight than for LBW and SGA; because of a greater biological susceptibility among babies born to mothers with lower exposures to other risk factors.

Another interpretation could be that additional adjustment for area income deprivation constitutes incorrect adjustment for a variable which is not a true confounder of the relationship of interest; in other words that it constitutes over-adjustment.^39^ In this study, we characterise exposure to areas with high crime rates as a proxy for a more general and latent ‘deprivation’ characteristic of neighbourhoods that may comprise a crime component but one that is also likely to constitute other characteristics of the local neighbourhood that are co-related to rates of recorded crime. In other words, we are arguing that of the typical candidate area measures that are often utilised, including the various domains of the SIMD, crime rates represents perhaps the most plausible in terms of an aetiological pathway between possible area effects and foetal growth and prematurity. Where individual level information is inadequate some studies adjust for area characteristics such as income deprivation in order to absorb variation from missing individual level effects. However, we would argue that, in order to capture the true magnitude of area effects, it is perhaps optimal to try and measure individual level effects rigorously at the individual level and not to have a crude mixture of both. Through record linkage used in this study, we were able to adjust for a range of individual level factors including smoking and a wide range of socio-economic status variables. Thus, it could be argued that omitting area income deprivation is a more robust analytical approach because unless we could identify a plausible aetiological pathway through which it might act on foetal growth or risk of prematurity, as *an independent area effect*, including it would constitute over-adjustment.

This study has limitations. Firstly, the available crime information was restricted to two years which were towards the end of births in the cohort and we therefore assume that crime levels remained relatively stable over time. However, if they do not, the resultant measurement error is most likely to be at random and therefore bias the effect towards the null. Secondly, uncertainty in the length of gestation which in our data, as in many others, was derived from the mother’s estimated date of last menstrual period may have introduced error in the models which will reduce the precision of resulting estimates.^40^

In conclusion, this study has found that adverse birth outcomes, particularly those related to foetal development, are associated with the rate of recorded crime in the mother’s area of residence at birth. This crime measure is likely to particularly reflect high levels of vandalism, minor assault and drug offences and therefore captures environments visibly laden with threat that elicit heightened fear of crime among residents.^31^ The findings were independent of a wide range of individual level effects including socio-economic status and smoking and area based measures of air pollution. The study therefore adds to the developing evidence base which asserts the potentially important role of the local social neighbourhood in determining spatial patterns in birth outcomes. It suggests that the existence of environments that convey threat, should be seen as a public health concern alongside the more obvious direct experience of drug use or physical assault.
